# Supplementary material for: The causal relationship between the human gut microbiota and pyogenic arthritis: a Mendelian randomization study
Source: Front Cell Infect Microbiol. 2024 Nov 26;14:1452480. doi: 10.3389/fcimb.2024.1452480 (PMC11629706; doi:10.3389/fcimb.2024.1452480)
Supplement: Supplementary file 1 [file DataSheet1.zip › mendelian test/ebi-a-GCST90027565.csv_forest.pdf]

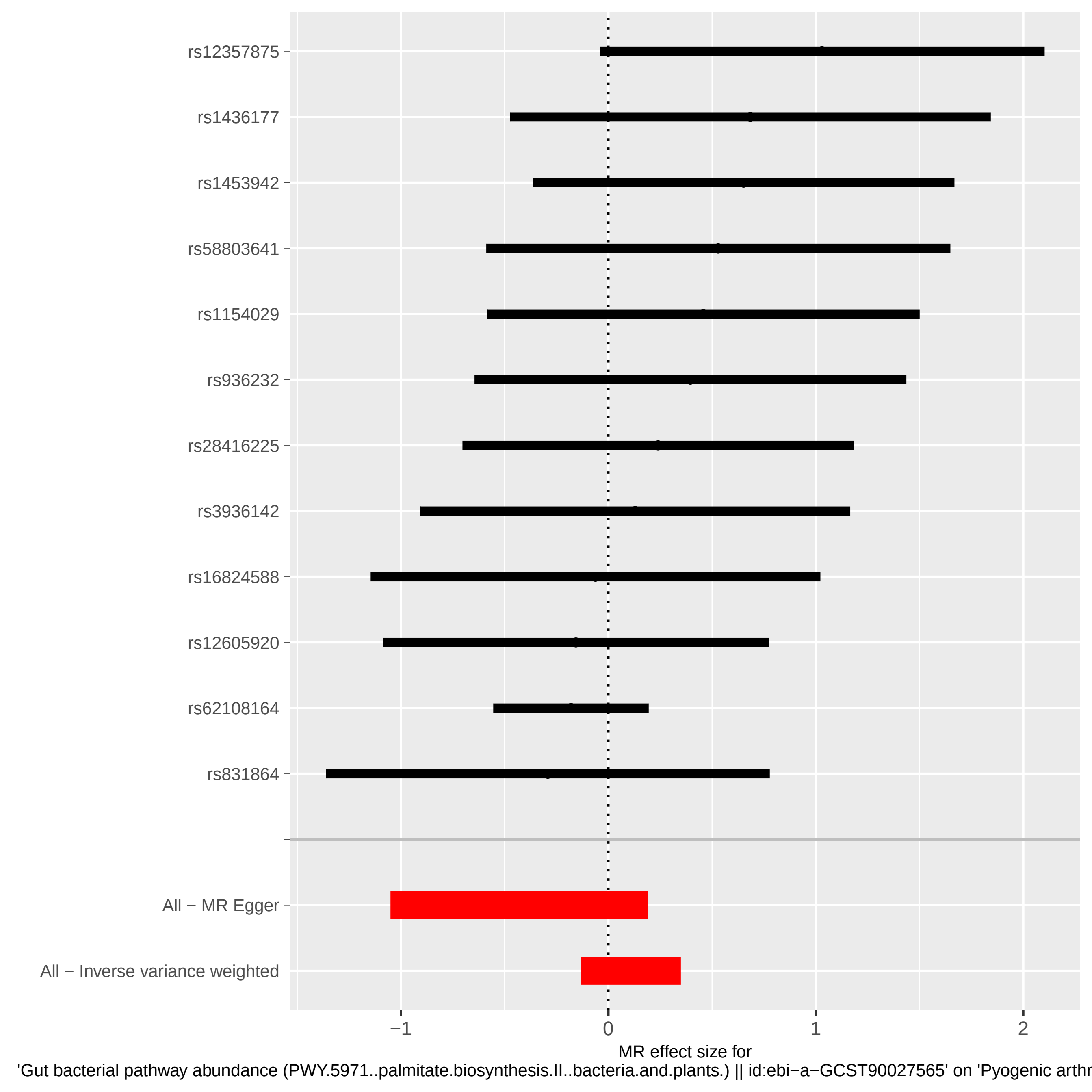
rs12357875
rs1436177
rs1453942
rs58803641
rs1154029
rs936232
rs28416225
rs3936142
rs16824588
rs12605920
rs62108164
rs831864
All − MR Egger
All − Inverse variance weighted
−1
0
1
2
MR effect size for
'Gut bacterial pathway abundance (PWY.5971..palmitate.biosynthesis.II..bacteria.and.plants.) || id:ebi−a−GCST90027565' on 'Pyogenic arthr
